# ﻿Morphological and phylogenetic analyses reveal two new species of *Rhodoveronaea* (Rhamphoriaceae, Rhamphoriales) from China

**DOI:** 10.3897/mycokeys.123.167930

**Published:** 2025-10-10

**Authors:** Hong Zhang, Ting-Hong Tan, Jian Ma

**Affiliations:** 1 School of Food Engineering, Moutai Institute, Renhuai, Guizhou 564507, China; 2 Guizhou Engineering Research Center for Health-Functional Liquor Brewing Technology, Moutai Institute, Renhuai, Guizhou 564507, China; 3 Special Food Resources Utilization Talent Base of Moutai Institute, Moutai Institute, Renhuai, Guizhou 564507, China; 4 School of Agriculture and Forestry Engineering and Planning, Tongren University, Tongren, Guizhou 554300, China; 5 Guizhou Provincial Key Laboratory for Biodiversity Conservation and Utilization in the Fanjing Mountain Region, Tongren University, Tongren, Guizhou 554300, China; 6 Center of Excellence in Fungal Research, Mae Fah Luang University, Chiang Rai 57100, Thailand; 7 Guizhou Industry Polytechnic College, Guiyang, Guizhou 550008, China; 8 School of Food and Pharmaceutical Engineering, Guizhou Institute of Technology, Guiyang, Guizhou 550003, China

**Keywords:** Asexual morph, phylogeny, Sordariomycetes, taxonomy, two new species

## Abstract

During a recent survey of freshwater fungi, four isolates were obtained from decaying wood in Chishui City, Guizhou Province, southern China. Based on phylogenetic analyses of a combined dataset (LSU, ITS, SSU, *tef1-α*, and *rpb*2) and detailed morphological comparisons, two novel species, *Rhodoveronaea aquisubtropica* and *R. guizhouensis*, are introduced. Comprehensive descriptions, illustrations, and results of the phylogenetic analyses supporting the taxonomic placement of these new taxa are provided. Additionally, a checklist of currently accepted *Rhodoveronaea* species supported by molecular data is included. Notably, *R. aquisubtropica* and *R. guizhouensis* represent the first records of *Rhodoveronaea* in the Chishui River Basin, thereby enriching the known diversity of subtropical freshwater habitats.

## ﻿Introduction

Freshwater fungi represent a taxonomically and ecologically diverse group of organisms that are widely distributed across various geographical regions, particularly in China (Guizhou, Hainan, Hong Kong, Taiwan, and Yunnan) and northern Thailand (Chiang Mai and Chiang Rai) (Zhang et al. 2011; Hyde et al. 2016, 2021; Su et al. 2016; Bao et al. 2018, 2019, 2020, 2021, 2023; Luo et al. 2019; Dong et al. 2020, 2021a, b; Fryar and Catcheside 2023; Fryar et al. 2023; Shen et al. 2024). They play a crucial role in the decomposition of organic matter and nutrient cycling within freshwater ecosystems (Luo et al. 2004; Hyde et al. 2016; Calabon et al. 2022, 2023). These fungi typically colonize fully or partially submerged woody substrates in a variety of freshwater habitats, including ponds, lakes, rivers, streams, and swamps, as well as artificial environments such as pools, reservoirs, dams, drainage ditches, and water-cooling towers (Lu et al. 2018; Xiao et al. 2023; Yang et al. 2023; Ma et al. 2024; Wang et al. 2024a, b; Xu et al. 2025).

*Rhodoveronaea* was established by Arzanlou et al. (2007) with *R. varioseptata* as the type species. *Rhodoveronaea* species are primarily known from their asexual morphs, which are characterized by reddish-brown, septate, straight or flexuous conidiophores with inflated basal cells, terminally integrated conidiogenous cells with crowded, slightly conspicuous conidium-bearing denticles, pale brown, ellipsoidal to obovoidal and septate conidia with a protruding base and a marginal basal frill (Arzanlou et al. 2007; Réblová 2009; Luo et al. 2019; Crous et al. 2021, 2023; Hyde et al. 2023; Chen et al. 2024; Yang et al. 2025). The first sexual morph characterized by immersed ascomata with subglobose to conical venter bearing conical neck, filamentous, septate paraphyses longer than asci, unitunicate, cylindrical asci with long-stipitate and fusiform, septate, hyalina ascospores (Réblová 2009). Currently, eight species are accepted within the genus *Rhodoveronaea*, which occur in both freshwater and terrestrial habitats (Chen et al. 2024; Yang et al. 2025).

In this study, four hyphomycetes isolates were obtained from freshwater habitats in Chishui River, Guizhou Province, China. Based on morphological characteristics, illustrations, and multi-gene phylogenetic analyses, two novel species are introduced, namely, *Rhodoveronaea aquisubtropica* and *R. guizhouensis*.

## ﻿Materials and methods

### ﻿Sample collection, examination, and isolation

Samples of submerged decaying wood pieces were collected from the Chishui River, Chishui City, Guizhou Province in southern China. Samples were taken to the laboratory in plastic bags with the collection details, including localities and dates (Rathnayaka et al. 2024). Fresh specimens were incubated in zip-lock bags and sterile, moist plastic boxes at room temperature for two weeks. The microscopic features were examined and photographed using a stereomicroscope (SMZ-168, Nikon, Japan) and an ECLIPSE Ni compound microscope (Nikon, Tokyo, Japan) with a Canon 90D digital camera. Measurements were made using Tarosoft (R) Image Frame Work software. Photo-plates were assembled using Adobe Photoshop CC 2019 (Adobe Systems, USA).

Single spore isolations were performed on PDA plates following the methods described by Chomnunti et al. (2014) and Senanayake et al. (2020), and the germinated conidia were aseptically transferred to fresh PDA plates. Morphological characters of fungal mycelium, including color, shape, and size, were documented. Dried fungal specimens were deposited in the Herbarium of Guizhou Academy of Agriculture Sciences (Herb. GZAAS), Guiyang, China. Pure cultures were deposited in the Guizhou Culture Collection, China (GZCC), Guiyang, China. The MycoBank numbers were obtained as described in https://www.mycobank.org/.

### ﻿DNA extraction, PCR amplification, and sequencing

Fresh fungal mycelia were scraped from colonies grown on PDA plates and transferred to a 1.5 mL microcentrifuge tube using a sterilized lancet for genomic DNA extraction. Genomic DNA was extracted using the Biospin Fungus Genomic DNA Extraction Kit (BioFlux, China). LR0R/LR5,ITS5/ITS4, NS1/NS4, EF1-983F/EF1-2218R, and fRPB2-5F/fRPB2-7cR were employed to amplify large ribosomal subunit (LSU; Vilgalys and Hester 1990), the internal transcribed spacer (ITS; White et al. 1990), small subunit of the nuclear ribosomal DNA (SSU, Rehner and Samuels 1994), translation elongation factor 1-α (*tef1-α*; Rehner and Buckley 2005), and RNA polymerase II second largest subunit (*rpb2*; Liu et al. 1999) sequence fragments, respectively. DNA preparation was conducted in a 50 μL mixture, which included 2 μL ofDNA, 2 μL of each forward and reverse primer, and 44 μL of 1.1 × T3 Supper PCR Mix (Qingke Biotech, Chongqing, China). The conditions for the polymerase chain reaction (PCR) correspond to those reported by Chen et al. (2024). The PCR products were purified and sequenced with the same primers at Beijing Tsingke Biotechnology Co., Ltd.

### ﻿Phylogenetic analyses

The newly obtained sequences were checked and assembled using BioEdit v.7.0.5.3 (Hall 1999) and SeqMan v.7.0.0 (DNASTAR, Madison, WI, USA; Swindell and Plasterer 1997), respectively. The sequences incorporated in this study were downloaded from GenBank (Table 1; https://www.ncbi.nlm.nih.gov/). Multiple sequences were aligned using MAFFT v.7.473 (https://mafft.cbrc.jp/alignment/server/; Katoh et al. 2019). The dataset was trimmed using trimAl v.1.2rev59 software (Capella-Gutiérrezet et al. 2009). A combined sequence dataset was created using SequenceMatrix-Windows-1.7.8 software (Vaidya et al. 2011).

**Table 1.** Taxa used in this study and their GenBank accession numbers.

Taxon	Strain	GenBank Accessions				
		LSU	ITS	SSU	tef1-α	rpb2
*Myrmecridium schulzeri*	CBS 100.54	EU041826	EU041769	-	-	-
*Myrmecridium sorbicola*	CBS 143433^T^	MH107948	MH107901	-	-	-
*Rhamphoria pyriformis*	CBS 139033	KT991665	KT991677	MG600406	-	KT991656
*Rhamphoria pyriformis*	CBS 139024	MG600397	MG600392	MG600405	-	MG600401
*Rhamphoriopsis aquimicrospora*	GZCC 20-0515^T^	OP377911	OP377812	OP377996	OP472992	OP473085
*Rhamphoriopsis hyalospora*	MFLU 19-2849^T^	MN846342	MN846344	-	-	-
*Rhamphoriopsis muriformis*	CBS 127683	MG600395	MG600389	MG600403	-	MG600400
*Rhamphoriopsis muriformis*	CBS 131269^T^	MG600396	-	MG600404	-	MG600399
*Rhamphoriopsis sympodialis*	HKAS 105172^T^	MT079191	MT079187	-	-	-
*Rhodoveronaea aquatica*	MFLUCC 18-1339^T^	MK849785	MK828641	MK828310	MN194046	-
*Rhodoveronaea aquatica*	GZCC 20-0447	OP377947	OP377862	OP378027	OP473041	OP473107
Rhodoveronaea aquisubtropica	GZCC 25-0635^T^	PX062375	PX062369	PX062379	PX072387	PX072391
Rhodoveronaea aquisubtropica	GZCC 24-0170	PX062376	PX062370	PX062380	PX072388	PX072392
*Rhodoveronaea everniae*	CBS 148309^T^	OK663776	OK664737	-	-	OK651172
Rhodoveronaea guizhouensis	GZCC 25-0633^T^	PX062373	PX062367	PX062377	PX072385	PX072389
Rhodoveronaea guizhouensis	GZCC 25-0634	PX062374	PX062368	PX062378	PX072386	PX072390
*Rhodoveronaea hainanensis*	GZAAS 22-2020^T^	OP748932	OP748935	-	-	-
*Rhodoveronaea hyalina*	GZCC 23-0622^T^	PP102207	PP102206	PP102214	PP259403	PP259399
*Rhodoveronaea lignicola*	GZCC 23-0624^T^	PP102209	PP102212	PP102216	PP259405	PP259401
*Rhodoveronaea nieuwwulvenica*	CBS 149447^T^	OQ629048	OQ628466	-	OQ627955	OQ627935
*Rhodoveronaea querci*	HKAS 145561^T^	PV097144	PQ932528	PV097142	PV164602	-
*Rhodoveronaea* sp.	XX-2025a^T^	-	PV053196	PV053387	-	-
*Rhodoveronaea varioseptata*	CBS 431.88^T^	MH873827	MH862134	-	-	-
*Rhodoveronaea varioseptata*	CBS 123472	FJ617559	MG600393	MG600408	-	JX066701
*Rhodoveronaea varioseptata*	CBS 123473	FJ617560	KT991676	JX066710	-	JX066700
*Xylolentia aseptata*	GZCC 20-0424^T^	OP377944	OP377859	OP378024	OP473038	OP473104
*Xylolentia aseptata*	GZCC 20-0426	OP377945	OP377860	OP378025	OP473039	OP473105
*Xylolentia brunneola*	PRA 13611^T^	MG600398	MG600394	MG600407	-	MG600402
*Xylolentia reniformis*	MFLU 19-0210^T^	MK547648	MK547646	-	-	-

Note: “^T^” indicates ex-type strains.Newly generated sequences are inbold. “-” indicates the unavailable data in GenBank.

Maximum likelihood (ML) analysis was carried out using the IQ Tree online website (http://iqtree.cibiv.univie.ac.at/) based on Bayesian Information Criteria (BIC) (Nguyen et al. 2015). The substitution model was automatically tested by the server. Bayesian Inference (BI) analysis was performed by using MrBayes on XSEDE (3.2.7a) via CIPRES (Stamatakis 2014). The aligned fasta file was converted to the nexus format file by using AliView (Daniel et al. 2010). The best-fit evolutionary model for individual dataset was determined using MrModeltest v. 2.3. 10 (Nylander et al. 2008). The GTR+G+I substitution model was selected for LSU, *tef1-α*, and *rpb*2, whereas the SYM+I+G and HKY+G models were applied to ITS and SSU, respectively.The posterior probabilities (BYPP) were determined based on Bayesian Markov chain Monte Carlo (BMCMC) sampling (Huelsenbeck and Ronquist 2001). Five simultaneous Markov chains were run for 10,000,000 generations, and trees were sampled every 1,000^th^ generation. The burn-in phase was set at 25%, and the remaining trees were used for calculating posterior probabilities (BYPP).

Trees were visualized using FigTree v.1.4.4 and edited with Adobe Illustrator CC 2019 (v.23.1.0; Adobe Systems, USA).

## ﻿Results

**Figure 1.**
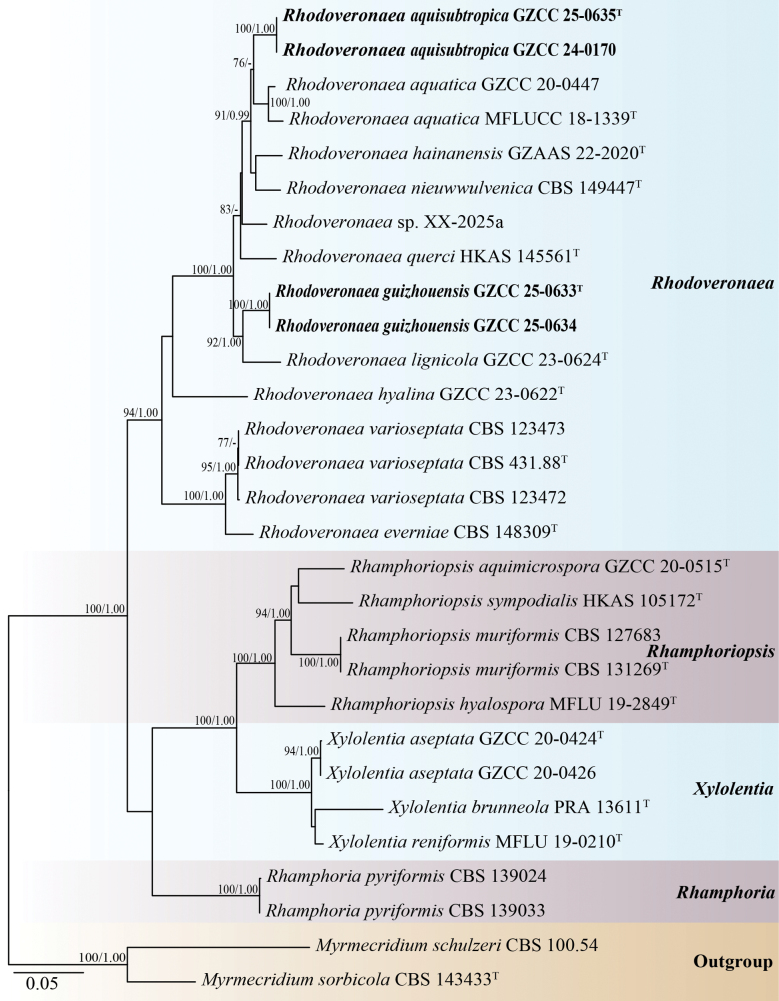
Phylogenetic tree generated from the RAxML analysis based on the combined LSU, ITS, SSU, *tef1-α*, and *rpb*2 sequence data. Bootstrap support values of RAxML (ML) equal to or greater than 75%, and Bayesian posterior probabilities (PP) equal to or greater than 0.95 are given near the nodes as ML/BYPP, respectively. The Maximum Likelihood (ML) and Bayesian Inference (BI) analyses yielded similar tree topologies. Hyphen (“-”) indicates a posterior probability lower than 0.95 for Bayesian.*Myrmecridium schulzeri* (CBS 100.54) and *M. sorbicola* (CBS 143433) were selected as outgroups. Ex-type strains are denoted with “^T^” and newly obtained isolates are in bold black fonts.

The phylogenetic placements of the four new strains were determined by multi-locus phylogenetic analysis. The concatenated sequence matrix comprised 4,318 characters (LSU: 1–860,ITS: 861–1,402, SSU: 1,403–2,409, *tef1-α*: 2,410–3,309, and *rpb*2: 3,310–4,318) across 29 taxa. Base frequencies and rates were A = 0.251521, C = 0.250418, G = 0.274699, and T = 0.223362; substitution rates were AC = 1.428534, AG = 2.806428, AT = 1.229261, CG = 1.263192, CT = 7.729689, and GT = 1.000000. The distribution shape parameter α equaled 0.144696. Fig. 1 presents the best-scoring ML tree, which had a final log-likelihood value of -16,381.027.

Based on the multi-gene phylogenetic tree (Fig. 1), our collections represent two species of *Rhodoveronaea* within the family Rhamphoriaceae. Our isolates, GZCC 24-0170 and GZCC 25-0635, group together and this clade forms a distinct lineage with *Rhodoveronaea aquatica* (GZCC 20-0447 and MFLUCC 18-1339) with 76% ML support. In addition, GZCC 25-0633 and GZCC 25-0634 form a clade together and are sister to *Rhodoveronaea lignicola* (GZCC 23-0624) with 92% ML and 1.00BYPP support.

### ﻿Taxonomy

#### ﻿ Rhodoveronaea aquisubtropica

Taxon classificationFungiRhamphorialesRhamphoriaceae

H. Zhang & J. Ma sp. nov.

CD56C901-161C-5128-B95F-D4A03F0794A6

904169

Fig. 2

##### Etymology.

“aqui-’’ refers to aquatic habitat of this fungus, and ‘‘-subtropica’’ means the climate type where the fungus was collected.

##### Holotype.

GZAAS 25-0665.

##### Description.

***Saprobic*** on decaying wood in a freshwater habitat. ***Sexual morph*** Undetermined. ***Asexual morph*** Hyphomycetous. ***Colonies*** on wood effuse, hairy, scattered or aggregated, brown. ***Mycelium*** partly superficial, partly immersed, composed of branched, septate, guttulate, smooth-walled, hyalina to brown hyphae. ***Conidiophores*** 204–270 × 4.9–7 μm (x̄ = 241.5 × 6 μm, n = 20), macronematous, mononematous, erect, flexuous, solitary, cylindrical, smooth-walled, septate, unbranched, black brown, mid brown, paler towards the apex. ***Conidiogenous cells*** polyblastic, integrated, terminal, determinate, sympodial, forming a rachis with subdenticulate loci, flexuous, pale brown to subhyalina, pigmented, with inconspicuous denticles. ***Conidia*** 10.5–13.5 × 4–5.5 μm (x̄ = 12.5 × 4.7 μm, n = 25), acropleurogenous, aggregated in slimy masses, ellipsoidal to narrowly obovoid, 1–3-septate, mostly 3-septate, smooth-walled, pale yellowish brown, guttulate, thin-walled, sometimes slightly constricted at the septa.

**Figure 2.**
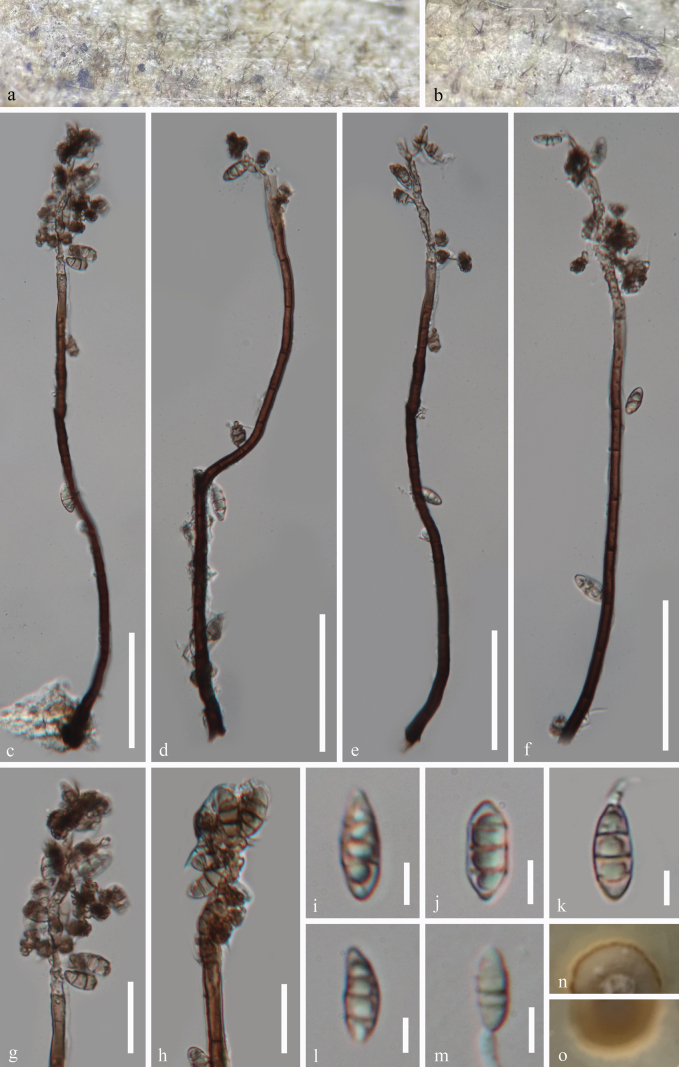
*Rhodoveronaea aquisubtropica* (GZAAS 25-0665, holotype). a, b. Colonies on the host surface; c–f. Conidiophores, conidiogenous cells and conidia; g, h. Conidiogenous cells and conidia; i–l. Conidia; m. Germinated conidium; n, o. Colonies on PDA from above and below after 36 days of incubation at room temperature. Scale bars: 50 μm (c–f); 20 μm (g, h); 5 μm (i–m).

##### Culture characteristics.

Conidia germinating on PDA within 12 hours, producing germ tubes from the conidial body. Colonies on PDA are circular with a raised surface and entire margin, reaching 30 mm in diameter after 36 days at room temperature (approximately 25 °C), and are brown to reddish brown in color on both the surface and reverse sides.

##### Material examined.

China • Guizhou Province, Chishui City, Chishui River, on decaying submerged wood in a freshwater habitat, 10 October 2024, Hong Zhang & Jian Ma, CS12 (GZAAS 25-0665, holotype), ex-type living cultures GZCC 25-0635; *Ibid*., CS18 (GZAAS 24-0083, paratype), living culture GZCC 24-0170.

##### Notes.

In our phylogenetic tree (Fig. 1), our isolates (GZCC 25-0635 and GZCC 24-0170) formed a sister clade to *Rhodoveronaea aquatica* (GZCC 20-0447 and MFLUCC 18-1339) with 76% ML support. A comparison of LSU, ITS, SSU, and *tef1-α* sequences between *Rhodoveronaea aquatica* (MFLUCC 18-1339) and *R. aquisubtropica* (GZCC 25-0635) and reveals their nucleotide differences of 3/774 bp (0.4%, without gap),20/502 bp (4%, including 4 gaps), 5/852 bp (0.6%, including 3 gaps), and 29/882 bp (3.3%, without gap), respectively, indicating that they are distinct species. Morphologically, *Rhodoveronaea aquisubtropica* (GZAAS 25-0665) differs from *R. aquatica* (MFLU 18-1593, ex-type) by its narrower conidiophores (204–270 × 4.9–7 μm*vs.*182–310 × 9–13 μm), and smaller conidia (10.5–13.5 × 4–5.5 μm vs. 23–27 × 9–11 μm) (Luo et al.2019). Therefore, we introduce *Rhodoveronaea aquisubtropica* as a novel species based on the multi-gene phylogenetic analysis and morphological differences.

#### ﻿ Rhodoveronaea guizhouensis

Taxon classificationFungiRhamphorialesRhamphoriaceae

H. Zhang & J. Ma sp. nov.

029CFEE4-3E6E-58A2-A1EC-243B40E6BD88

904170

Fig. 3

##### Etymology.

The specific epithet ‘*guizhouensis*’ refers to the locality “Guizhou Province”, from where the holotype was collected.

##### Holotype.

GZAAS 25-0663.

##### Description.

***Saprobic*** on decaying submerged wood in a freshwater habitat. ***Sexual morph*** Undetermined. ***Asexual morph*** Hyphomycetous. ***Colonies*** on wood effuse, hairy, scattered or aggregated, brown. ***Mycelium*** partly superficial, partly immersed, composed of branched, septate, guttulate, smooth-walled, hyalina to brown hyphae. ***Conidiophores*** 211–268 × 4.5–6.3 μm (x̄ = 234 × 5.2 μm, n = 25), macronematous, mononematous, erect, flexuous, solitary, cylindrical, smooth-walled, septate, unbranched, black brown, mid brown, paler towards the apex. ***Conidiogenous cells*** polyblastic, integrated, terminal, determinate, sympodial, forming a rachis with subdenticulate loci, flexuous, pale brown to subhyalina, pigmented, with inconspicuous denticles. ***Conidia*** 11.5–17 × 4.3–6 μm (x̄ = 13.8 × 5.2 μm, n = 25), acropleurogenous, aggregated in slimy masses, ellipsoidal to narrowly obovoid, 1–3-septate, mostly 3-septate, smooth-walled, pale yellowish brown, guttulate, thin-walled, sometimes slightly constricted at the septa.

##### Culture characteristics.

Conidia germinating on PDA within 11 hours, producing germ tubes from the conidial body. Colonies on PDA are circular with a raised surface and entire margin, reaching 27 mm in diameter after 32 days at room temperature (approximately 25 °C), and are white, reddish brown to black in color on both the surface and reverse sides.

**Figure 3.**
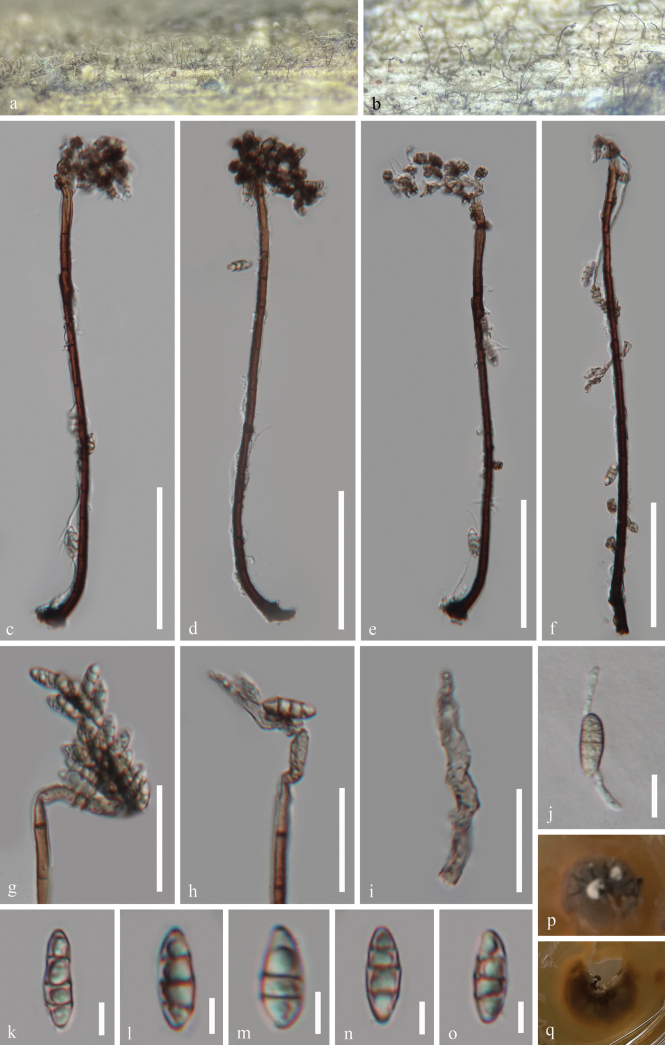
*Rhodoveronaea guizhouensis* (GZAAS 25-0663, holotype). a, b. Colonies on the host surface; c–f. Conidiophores, conidiogenous cells, and conidia; g–i. Conidiogenous cells and conidia; j. A germinated conidium; k–o. Conidia; p, q. Colonies on PDA from above and below after 32 days of incubation at room temperature. Scale bars: 60 μm (c–f); 30 μm (g, h); 20 μm (i); 10 μm (j); 5 μm (k–o).

##### Material examined.

China • Guizhou Province, Chishui City, Chishui River, on decaying submerged wood in a freshwater habitat, 10 October 2024, Hong Zhang &Jian Ma, CSF2 (GZAAS 25-0663, holotype), ex-type living cultures GZCC 25-0633; *Ibid*., CSF9 (GZAAS 25-0664, paratype), living culture GZCC 25-0634.

##### Notes.

In our phylogenetic tree (Fig. 1), our isolates (GZCC 25-0633 and GZCC 25-0634) formed a sister clade to *Rhodoveronaea lignicola* (GZCC 23-0624) with 92% ML and 1.00BYPP support. *Rhodoveronaea guizhouensis* (GZAAS 25-0663) can be distinguished from *R. lignicola* (GZAAS 23-0612) by its longer conidiophores (211–268 μm*vs.*75–125 μm), longer conidia (up to 17 μm*vs.*9–13.5 μm) (Chen et al. 2024). Moreover, base pair comparison of *Rhodoveronaea guizhouensis* (GZCC 25-0633) and *R. lignicola* (GZCC 23-0624) shows 30/528 bp differences in ITS (5.7%, gaps 5 bp), 7/862 bp differences in LSU (0.8%, gaps 2 bp), 3/910 bp differences in SSU (0.3%, without gap), 39/887 bp differences in *tef1-α* (4.4%, without gap), and 69/803 bp differences in *rpb*2 (8.6%, without gap). Therefore, based on DNA molecular data and morphological characteristics, we introduce *Rhodoveronaea guizhouensis* as a novel species.

## ﻿Discussion

The genus *Rhodoveronaea* currently comprises ten species, including the two new species described in the present study (Arzanlou et al. 2007; Luo et al. 2019; Crous et al. 2021, 2023; Hyde et al. 2023; Chen et al. 2024; Yang et al. 2025). Among them, four species are found in freshwater habitats and six in terrestrial habitats (Table 2), with primary distributions in Guizhou, Yunnan, and Hainan provinces of China.*Rhodoveronaea* species are distributed in China, Czech Republic, France, Germany, Netherlands, and Sweden (Arzanlou et al. 2007; Réblová 2009; Luo et al. 2019; Crous et al. 2021, 2023; Hyde et al. 2023; Chen et al. 2024; Yang et al. 2025). They occur as saprobes on bamboo stick, *Bertia moriformis*, *Carpinus betulus*, *Evernia prunastri*, and decaying wood in both freshwater and terrestrial habitats (Arzanlou et al. 2007; Réblová 2009; Luo et al. 2019; Crous et al. 2021, 2023; Hyde et al. 2023; Chen et al. 2024; Yang et al. 2025).

**Table 2.** A checklist of accepted *Rhodoveronaea* species with molecular data.

Species	Distribution	Habitat	Reference
*Rhodoveronaea aquatica*	China	Freshwater	Luo et al. (2019); Yang et al. (2023)
Rhodoveronaea aquisubtropica	China	Freshwater	This study
*Rhodoveronaea everniae*	Netherlands	Terrestrial	Crous et al. (2021)
Rhodoveronaea guizhouensis	China	Freshwater	This study
*Rhodoveronaea hainanensis*	China	Freshwater	Hyde et al. (2023)
*Rhodoveronaea hyalina*	China	Terrestrial	Chen et al. (2024)
*Rhodoveronaea lignicola*	China	Terrestrial	Chen et al. (2024)
*Rhodoveronaea nieuwwulvenica*	Netherlands	Terrestrial	Crous et al. (2023)
*Rhodoveronaea querci*	China	Terrestrial	Yang et al. (2025)
*Rhodoveronaea varioseptata*	Czech Republic, France, Sweden	Terrestrial	Arzanlou1 et al. (2007); Réblová 2009

Note: The newly isolated species in this study are highlighted in bold.

Based on multi-gene phylogenetic analyses, considerable morphological variation can occur even within the same *Rhodoveronaea* species. For example, two collections (MFLU 18-1593 from decaying submerged wood in Yunnan Province and HKAS 112574 from a similar substrate in Guizhou Province, China) have both been identified as the same species, namely *Rhodoveronaea aquatica* (Luo et al. 2019; Yang et al. 2023). However, MFLU 18-1593 exhibits significantly larger conidiophores (182–310 × 9–13 μm) and longer conidia (23–27 × 9–11 μm) compared to those of HKAS 112574 (conidiophores: 70–147 × 3–4.5 μm; conidia: 10–13 × 4–6 μm) (Luo et al. 2019; Yang et al. 2023).

The two *Rhodoveronaea* species, *R. aquisubtropica* and *R. guizhouensis*, isolated from the Chishui River, exhibit highly similar morphological characteristics. However, multi-gene phylogenetic analyses (Fig. 1) provide strong support for their recognition as distinct species. This divergence may be attributed to molecular evolutionary processes, during which the two species independently developed convergent morphological traits in response to comparable environmental conditions. These findings highlight the limitations of morphology-based taxonomy alone and emphasize the importance of molecular data for accurate species delimitation. Moreover, this study underscores the potential for discovering novel fungal taxa in riverine ecosystems, particularly in ecologically rich yet underexplored regions.

## Supplementary Material

XML Treatment for Rhodoveronaea aquisubtropica

XML Treatment for Rhodoveronaea guizhouensis

## ﻿Additional information

### Use of AI

No use of AI was reported.

### Author ORCIDs

Hong Zhang https://orcid.org/0000-0003-1894-8169

Ting-Hong Tan https://orcid.org/0000-0002-2933-2696

Jian Ma https://orcid.org/0009-0008-1291-640X

### Data availability

All of the data that support the findings of this study are available in the main text.
